# Effects of different cultivation methods on the rhizosphere microbial community and secondary metabolites of *Houttuynia cordata* Thunb.

**DOI:** 10.7717/peerj.20797

**Published:** 2026-02-11

**Authors:** Fangmei Song, Die Fu, Anping Wang, Zhannan Yang, Tianhua Yu

**Affiliations:** Key Laboratory for Information System of Mountainous Areas and Protection of Ecological Environment of Guizhou Province, Guizhou Normal University, Guiyang, China

**Keywords:** Plant-microbe interactions, Community structure, Medicinal plant cultivation, Environmental drivers

## Abstract

This study aimed to investigate the effects of three distinct cultivation methods on the plant-soil system of *Houttuynia cordata* Thunb. specifically focusing on how they shape the rhizosphere microbial community and influence the accumulation of it is phenolic compounds. This study employed high-throughput sequencing of bacterial 16S rDNA and fungal ITS rDNA to assess the impact of three cultivation methods including *in situ* cultivation (ISC), indoor cultivation (IC), and tissue culture (TC) on the diversity and community structure of *H. cordata* rhizosphere soil microbes. Additionally, we explored the environmental drivers of phenotypic variations in secondary metabolite composition. Soil pH, urease (URA), total potassium (TK), and total nitrogen (TN) were significantly correlated with the accumulation of quercitrin, kaempferol-3-O-glucorhamnoside, isoquercitrin, and chlorogenic acid in *H. cordata*. Moreover, these environmental factors significantly influenced the rhizosphere microbial taxa * Saitozyma*, * Lysobacter*, * Gemmata*, and * Penicillium*. IC presents a sustainable approach for *H. cordata* cultivation, enhancing rhizosphere soil fertility and health. Furthermore, pH, URA, TK, and TN serve as key environmental drivers of secondary metabolite variation. These findings provide a foundation for establishing quality evaluation standards for *H. cordata* ensuring stable pharmacological efficacy, and facilitating further functional applications.

## Introduction

*Houttuynia cordata* Thunb. (Saururaceae) is widely distributed across Southeast and South Asian countries, particularly in Myanmar, India, Thailand, and Vietnam. In China, it is predominantly found in the southern regions, particularly in the Yangtze River Basin and its southern provinces, including Yunnan, Guizhou, Hunan, Sichuan, and Chongqing, where it exhibits strong adaptability (Liu et al., 2021). *H. cordata* is a perennial herb in the Saururaceae family, is valued in traditional Chinese medicine for its medicinal and edible properties. The entire plant is widely cultivated in China and is commonly utilized in dietary supplements and herbal medicine, contributing significantly to its economic value (Chinese Pharmacopoeia Commission, 2020). It is reported that essential oils extracted from *H. cordata* exhibit a distinctive fishy odor and possess various bioactive properties. The plant is rich in flavonoids, volatile oils, phenolic acids, and alkaloids, making it a medicinal and edible plant with immunomodulatory, antioxidant, antibacterial, laxative, diuretic, stomachic, antiviral, anti-inflammatory, anthelmintic, and immune-regulatory effects (Liu et al., 2024). Chlorogenic acid and flavonoids are the two primary bioactive components of *H. cordata*, demonstrating potent radical scavenging, antioxidant, antipyretic, antibacterial, anticancer, and antimutagenic activities (Rebícková et al., 2020).

The accumulation of plant secondary metabolites is a highly complex network, co-regulated by the intrinsic genetic regulatory network and external environmental stimuli (Li et al., 2020). In wild populations, this complexity is particularly pronounced because the wild environment exhibits high heterogeneity, leading plants to face highly variable conditions in climate, soil, moisture, light, and biotic stresses (Li et al., 2020; Yang et al., 2018; Qaderi, Martel & Strugnell, 2023). Plants of different genotypes exhibit significant differences in the types, content, and biosynthetic efficiency of their secondary metabolites (Li et al., 2020; Yang et al., 2018). Driven by long-term natural selection and environmental adaptation, wild *H. cordata* populations may diverge into distinct genotypes, which in turn can lead to differences in their synthetic capacity for metabolites such as flavonoids, volatile oils, and phenolic acids (Li et al., 2020). For example, a study on dandelion revealed that the composition and content of root secondary metabolites exhibit significant genotype × environment (G×E) interactions under different climatic conditions (Bont et al., 2020). This underscores the important role of genetic factors in the accumulation of adaptive metabolites. Thus, even when growing in the same wild habitat, individual *H. cordata* plants of different genotypes may still display distinct profiles and concentrations of secondary metabolites (Yang et al., 2018). Various abiotic and biotic factors in the wild impose significant regulatory effects on the synthesis and accumulation of plant secondary metabolites (Li et al., 2020; Yang et al., 2018; Qaderi, Martel & Strugnell, 2023). Extreme temperatures, drought, or high humidity can alter the metabolic pathways in *H. cordata*, thereby promoting or inhibiting the synthesis of specific secondary metabolites (Qaderi, Martel & Strugnell, 2023; Espinosa-Leal et al., 2022). In *H. cordata* growing under forest canopies, insufficient light due to shading may reduce carbon assimilation products, which in turn can restrict the synthesis of secondary metabolites (Li et al., 2020; Yang et al., 2018). Conversely, ample sunlight may promote the accumulation of certain light-sensitive secondary metabolites. In wild *H. cordata*, the accumulation of these compounds ultimately represents the complex interplay between its genotype and diverse environmental factors (Li et al., 2020; Yang et al., 2018). This implies that even among wild *H. cordata* individuals with similar genetic backgrounds, those inhabiting different micro-environments (such as varying slope aspects, altitudes, and soil types) may still exhibit distinct secondary metabolite profiles (Li et al., 2020; Yang et al., 2018).

It is reported that the phenotypic traits, functional characteristics, and secondary metabolite content of medicinal plants are jointly determined by their growing environments (*e.g.*, geographical, climatic, and soil conditions) and their genotypes. Variation in these traits, particularly in the content of secondary metabolites, can lead to inconsistencies in the quality of herbal medicines (Yang et al., 2024). As an important medicinal species, *H. cordata* is no exception. It is wild populations that exhibit remarkable phenotypic plasticity in their natural habitats, which is primarily attributed to their genetic characteristics and the well-documented genotype × environment (G×E) interactions that influence secondary metabolite production. In wild populations, it is not feasible to isolate or control the profound impact of their inherent genetic background on secondary metabolite composition. However, conventional research methods find it difficult to fully eliminate the interference from these complex environmental factors, thereby limiting our ability to accurately decipher the drivers behind the secondary metabolic variation in *H. cordata*. Thus, under the premise of acknowledging and accounting for the uncontrollable nature of wild genotypes, elucidating the core mechanisms that drive its phenotypic differences—thereby providing a theoretical basis for enhancing secondary metabolite content, refining quality evaluation standards, stabilizing efficacy, and promoting sustainable utilization, remains a significant scientific challenge.

Microorganisms are essential for nutrient cycling, breaking down organic and inorganic matter to sustain biological processes. Microorganisms shape *H. cordata* production and quality by regulating it is secondary metabolites, influencing growth and post-harvest stages. Furthermore, microbial distribution and diversity can directly affect the quality of *H. cordata* (*e.g.*, herbal efficacy and edible taste). The plant rhizosphere represents the most immediate soil-plant interface, where root metabolic activities influence soil properties, facilitating interactions among plants, microbes, and soil. These interactions regulate plant growth and development, water and nutrient uptake, microbial survival and proliferation, and plant responses to environmental stresses (Bending et al., 2024).

Rhizosphere microorganisms are the most active life forms in the soil surrounding plant roots and play a critical role in material recycling and energy metabolism. They play key roles in organic matter degradation, humus formation, and nutrient cycling, serving as an integral part of the plant’s “second genome” (Bakker & Berendsen, 2022). Rhizosphere microbial community assembly depends on interactions between microbes, host plants, and environmental factors. These microbial communities exist in dynamic flux, forming intricate networks that shape the rhizosphere microbiome. Studies have demonstrated that soil physicochemical properties at a macro level influence microbial community structures, while plant species and developmental stages determine the selective enrichment of specific microbial taxa within the rhizosphere. Plant-rhizosphere microbe interactions span mutualism to antagonism, varying by plant species and environmental context. Disparities in microbial community structure between rhizosphere and non-rhizosphere soils are primarily attributed to strong plant-driven selection or microbial competition (Tian et al., 2020). Research on *Sorghum bicolor* under drought stress has revealed that sorghum exerts a strong selective effect on the soil fungal species pool (Gao et al., 2020). Rhizosphere microbes directly and indirectly affect plant growth and stress tolerance (Parasar, Sharma & Agarwala, 2024). Rhizosphere soil fungal communities are vital indicators of ecosystem health, as their genetic and functional diversity drives plant-soil-microbe interactions. Given these insights, we hypothesize that different cultivation methods may influence the structural composition of the *H. cordata* rhizosphere microenvironment. However, the mechanisms by which rhizosphere microbes regulate changes in *H. cordata* secondary metabolism remain unclear.

This study aims to determine the key factors driving variations in *H. cordata* secondary metabolite composition and elucidate whether these variations are primarily controlled by intrinsic genetic factors or external environmental conditions. Additionally, we seek to clarify changes in soil properties and microbial communities associated with *H. cordata* cultivation and explore the environmental drivers underlying secondary metabolite variations.

The objectives of the study are: (1) Analyze the rhizosphere microbial community structure of *H. cordata* under *in situ* cultivation (ISC) and indoor cultivation (IC) conditions, (2) compare differences in rhizosphere microbial diversity between ISC and IC, (3) identify environmental drivers influencing variations in *H. cordata* secondary metabolite composition and (4) elucidate the mechanisms by which indoor cultivation enhances *H. cordata* quality.

## Materials & Methods

### Sample collection and processing

**Figure 1:**
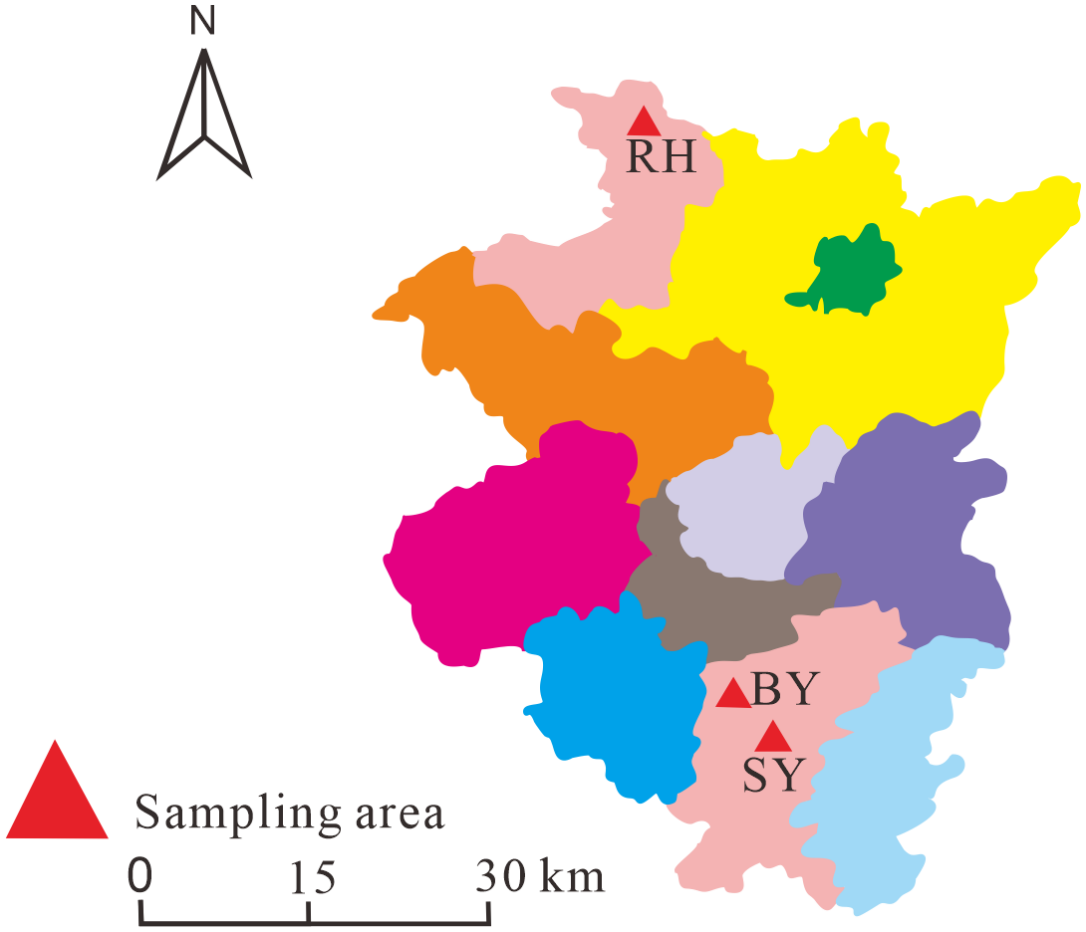
Map of *Houttuynia cordata* Thunb. collection showing sampling locations. Note: “RH” denotes the sampling site in Renhuai city, Guizhou province; “BY” denotes the sampling site in Baiyun district, Guizhou province; “SY” denotes the laboratory cultivation base in Guiyang city, Guizhou province.

In early March 2018, wild *H. cordata* samples were collected from two mountainous regions in Guizhou Province, China: Baiyun District, Guiyang City (26°41′56″N, 106°37′26″E) and Daba Town, Renhuai City (28°03′40″N, 106°24′36″E). At both sampling sites, a portion of *H. cordata* plants was left to grow naturally, representing the ISC group. Freshly collected *H. cordata* plants from both sites were transported to an indoor experimental base (26°35′31″N, 106°43′04″E) for transplantation, constituting the IC group. Additionally, bulk soil from the experimental base was collected before planting *H. cordata and* rhizosphere soil samples from ISC and IC groups were collected (Fig. 1). In late August 2018, three soil samples were taken per treatment. At each site, rhizosphere soil within 2.5 mm of healthy plant roots was collected. Samples were immediately sealed in sterile bags, chilled during transport, and stored at 4 ^∘^C until analysis. To obtain rhizosphere soil samples, sterile brushes were used to gently remove soil adhering to *H. cordata* roots. The soil was split into two parts; one was air-dried for physicochemical analysis, including pH and enzyme activity assays, while the other portion was stored at −80 °C for microbial community analysis using high-throughput sequencing of 16S rDNA (bacteria) and ITS rDNA (fungi).

### Experimental control

To identify the key drivers of secondary metabolite accumulation in *H. cordata* plants from the same source were subjected to three different cultivation methods: tissue culture (TC), IC, and ISC. The phenolic compound content of *H. cordata* was analyzed under each cultivation condition. By comparing the phenolic content in *H. cordata* grown under different cultivation methods, we aimed to differentiate whether variations in secondary metabolite accumulation were driven by environmental factors (*e.g.*, geographical conditions) or by intrinsic metabolic processes of the plant itself. The specific details of the three cultivation methods are presented in Table 1.

**Table 1:** Three cultivation models for the controlled experiment.

Cultivation method	Cultivation conditions	Code
Tissue culture	Fresh *H. cordata* samples were brought back to the laboratory. Young underground stem nodes were used as explants and sterilized with 75% medical ethanol for 30 s followed by 0.1% mercuric chloride solution for 10 min. The shoot induction medium contained 2 mg/L KT, 0.8% agar, and 3% sucrose in Murashige and Skoog (MS) medium, while the robust seedling culture medium contained 2 mg/L KT, 0.8% agar, and 3% sucrose in MS medium. The cultivation conditions: daytime temperature 27 ± 1 °C, nighttime temperature 25 ± 1 °C, photoperiod 12 h/day, and light intensity of 3,000 lx. Cultivation was conducted in a BIC intelligent artificial climate chamber.	TC
Indoor cultivation	Fresh *H. cordata* samples were brought back to the laboratory and young underground stem nodes were planted at the Mountain Environment Key Laboratory planting base of Guizhou Normal University (106°43′E, 26°35′N). No fertilizers were applied during the cultivation period, and regular watering was maintained.	IC
*In situ* cultivation	A portion of *H. cordata* was left to grow naturally at the sampling sites.The sampling site in Daba Town, Renhuai, Guizhou Province, has a mid-subtropical humid monsoon climate, characterized by distinct seasons, synchronized rainfall and heat, an average altitude of 880 m, an annual meantemperature of 16 °C, and an annual precipitation of 800–1,000 mm.	ISC
	The sampling site in Baiyun District, Guiyang City, falls within the subtropical climate zone, with strong alternation of cold and warm airflows, a pronounced monsoon plateau climate, no extreme summer heat, and no severe winter cold. The annual mean temperature is approximately 14 °C, with an annual precipitation of 1,147–1,191 mm. The altitude ranges from 1,140 to 1,618.5 m. Samples were collected in August after natural growth.

**Notes.**

The above are three cultivation conditions for *Houttuynia cordata* Thunb. including cultivation conditions, growth temperature, geographical location, climate, altitude, *etc*.

To minimize the confounding effects of external environmental variations while investigating the influence of different cultivation methods on *H. cordata* rhizosphere microbes, we conducted sampling along the same longitudinal gradient and employed three cultivation methods: *in situ* cultivation (ISC), indoor cultivation (IC), and tissue culture (TC). High-throughput sequencing on the Illumina HiSeq 2500 platform was employed to compare and characterize rhizosphere microbial community structure and diversity across different cultivation conditions. Concurrently, soil physicochemical properties were analyzed using soil chemistry methods to identify microenvironmental factors influencing bacterial and fungal communities in *H. cordata* rhizosphere soil across cultivation methods.

### Determination of phenolic content

Phenolic content in *H. cordata* was measured by HPLC following methanol ultrasonic extraction (Wang et al., 2024). The chromatographic conditions were as follows: the mobile phase consisted of an organic phase (acetonitrile: methanol = 11:5, v/v) and an aqueous phase 0.1% (formic acid solution) in a gradient elution mode. The chromatographic column used was a Shim-pack CLC-ODS(150  × 6.0 mm I.D, No: 61529098B), and the column temperature was maintained at 40 °C.

### Determination of soil physicochemical properties

The determination of basic soil nutrient elements followed standard analytical methods (Akhtar et al., 2018), including the measurement of soil pH, soil organic matter (SOM), total nitrogen (TN), total phosphorus (TP), total potassium (TK), alkali-hydrolyzable nitrogen (AN), available phosphorus (AP), and readily available potassium (AK). Three sets of soil samples were analyzed in this study, each with three replicates. Analytical methods included the glass electrode method for pH and potassium dichromate oxidation with external heating for organic matter, Kjeldahl digestion-alkaline diffusion method for TN, sodium hydroxide fusion-molybdenum-antimony anti-spectrophotometry for TP, sodium hydroxide glass electrode method for TK, sulfuric acid-potassium dichromate fusion-flame photometry for AN, alkaline diffusion-boric acid absorption method for AP, sodium bicarbonate extraction-molybdenum-antimony anti-spectrophotometry for available phosphorus, and ammonium acetate extraction-flame photometry for AK.

### Determination of soil enzyme activities

Soil enzyme activities were measured following established analytical methods from previous studies (Liu et al., 2023; Zhang, Zhang & Miao, 2023). Urease, sucrase, and phosphatase activities were quantified by incubating 1 g of soil at 37 °C for 24 h. Urease activity was expressed as the micrograms of ammonium nitrogen released, sucrase activity as the milligrams of glucose produced, and phosphatase activity as the milligrams of phenol generated.

### Genomic DNA extraction and polymerase chain reaction amplification

Rhizosphere soil samples of *H. cordata* were frozen at −80 °C upon collection, then transported on dry ice to Shanghai Baiqu Biotechnology Co., Ltd. for DNA extraction using the Soil DNA Kit. DNA integrity was checked by 1% agarose gel electrophoresis, and purity was measured. The V3—V4 region of the 16S rRNA gene was amplified using primers 341F (CCTACGGGNGGCWGCAG) and 806R (GGACTACHVGGGTATCTAAAT). The ITS2 region of the ITS rRNA gene was amplified using the primer pair KYO2F (GATGAAGAACGYAGYRAA) and ITS4R (TCCTCCGCTTATTGATATGC). Polymerase chain reaction (PCR) amplification was conducted under the following conditions: initial denaturation at 98 °C for 3 min, followed by 27 cycles of denaturation at 98 °C for 30 s, annealing at 50 °C for 30 s, and extension at 72 ° C for 30 s, with a final extension at 72 °C for 5 min and storage at 4 °C. The PCR products were purified using the AxyPrep DNA Gel Extraction Kit, and quantification was performed using a QuantiFluor™ fluorometer. The purified amplicons were pooled in equimolar amounts, ligated with sequencing adapters, and used to construct sequencing libraries. High-throughput sequencing was performed on the Illumina HiSeq 2500 platform.

### Bioinformatics and statistical analysis

The peak areas of various compounds in the high-performance liquid chromatography (HPLC) chromatograms of both control and sample groups were recorded. The concentrations of six phenolic compounds under different culture conditions were calculated using the external standard single-point method. Data were processed and visualized using Excel and other software, while statistical analyses were conducted using SPSS 19.0. Analysis of variance (ANOVA) was performed, with a significance level set at *P* < 0.05.

For soil 16S rDNA and ITS rDNA sequencing, raw reads were obtained and subjected to quality filtering to remove low-quality reads, followed by sequence assembly and further filtering. A 97% similarity threshold was applied to cluster sequences into operational taxonomic units (OTUs), ensuring the effective use of sequencing data. Alpha (*α*) and beta (*β*) diversity indices were analyzed using Mothur software. The *α*-diversity analysis included multiple indices. Using key alpha-diversity metrics, including the number of observed features, the Chao1 index, the Shannon index, and the Simpson index, to characterize the microbial community structure. Principal coordinate analysis (PCA) was conducted using R software to evaluate sample distances. Redundancy Discriminant Analysis (RDA) examined the relationship between rhizosphere microbial communities and soil environmental factors (Leng et al., 2023). All analyses included blank and parallel control experiments to ensure data reliability.

### Data processing

All experiments were conducted in triplicate. Data were processed and visualized using Excel and additional software. Statistical analyses were performed using SPSS 19.0, with analysis of variance (ANOVA) applied and a significance threshold set at *P* < 0.05. High-throughput sequencing data from soil samples were analyzed and visualized using the Microbiome Cloud Platform (WeGene Bioinformatics). Further graphical refinements and adjustments were performed using Origin 2021 and Adobe Illustrator.

## Results

### Effects of three cultivation methods on the six phenolic compounds in *H. cordata*

**Figure 2:**
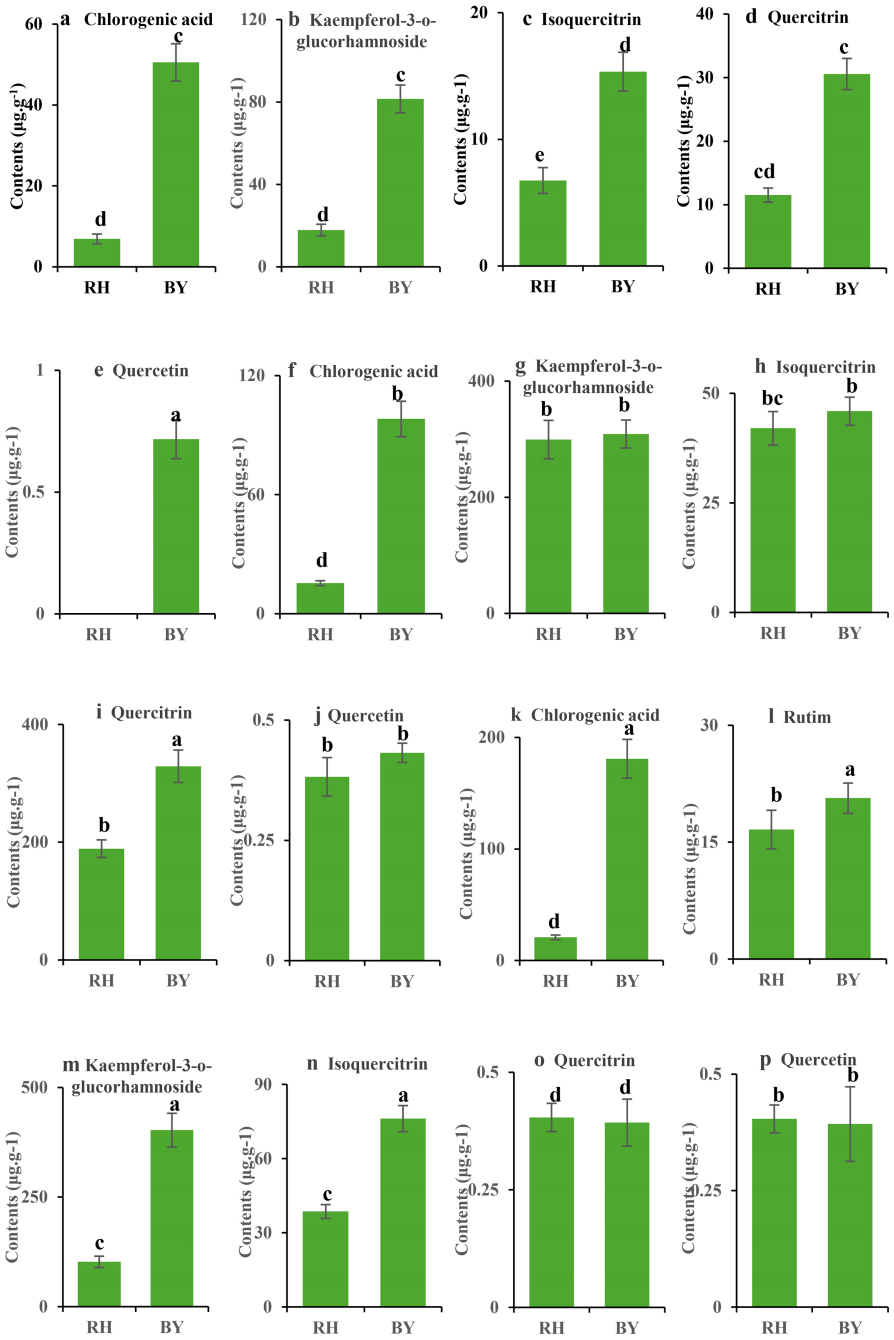
Comparison of six phenolic compound contents in *Houttuynia cordata* Thunb. under three cultivation methods: TC (A–E); IC (F–J); ISC (K–P). Note: “RH” indicates that the *H. cordata* samples were collected from a wild site in Renhuai city, Guizhou province; “BY” indicates that the samples were collected from a wild site in Baiyun district, Guizhou province. “TC”, “IC”, and “ISC” are abbreviations for tissue culture, indoor cultivation, and *in situ* cultivation, respectively.

The contents of six phenolic compounds in *H. cordata*, including chlorogenic acid, rutin, kaempferol-3-o-glucorhamnoside, isoquercitrin, quercitrin, and quercetin, were determined using HPLC. The results showed significant differences in the accumulation of these bioactive compounds among the three cultivation methods (Fig. 2). The chlorogenic acid content followed the order: ISC > IC > TC, with significantly higher levels in *H. cordata* from the BY site than from the RH site. rutin was not detected in TC or IC, but its content ranged from 16.6 to 20.6 µg/g in ISC. The contents of kaempferol-3-o-glucorhamnoside and isoquercitrin varied across the three cultivation methods, with the lowest kaempferol-3-o-glucorhamnoside levels observed in TC. *H. cordata* from the BY site had significantly higher kaempferol-3-o-glucorhamnoside levels than those from the RH site. quercitrin content was lowest in ISC, followed by TC, with the highest levels observed in IC. The BY site exhibited significantly higher quercitrin content than the RH site, except for ISC, where the levels were similar between the two sites. The overall quercetin content in *H. cordata* was relatively low, with minimal variation among cultivation methods. These findings suggest that the metabolite composition of *H. cordata* is strongly influenced by environmental factors. Among the three cultivation methods, the total phenolic compound content followed the trend: IC> ISC >TC.

### Soil physicochemical properties

**Figure 3:**
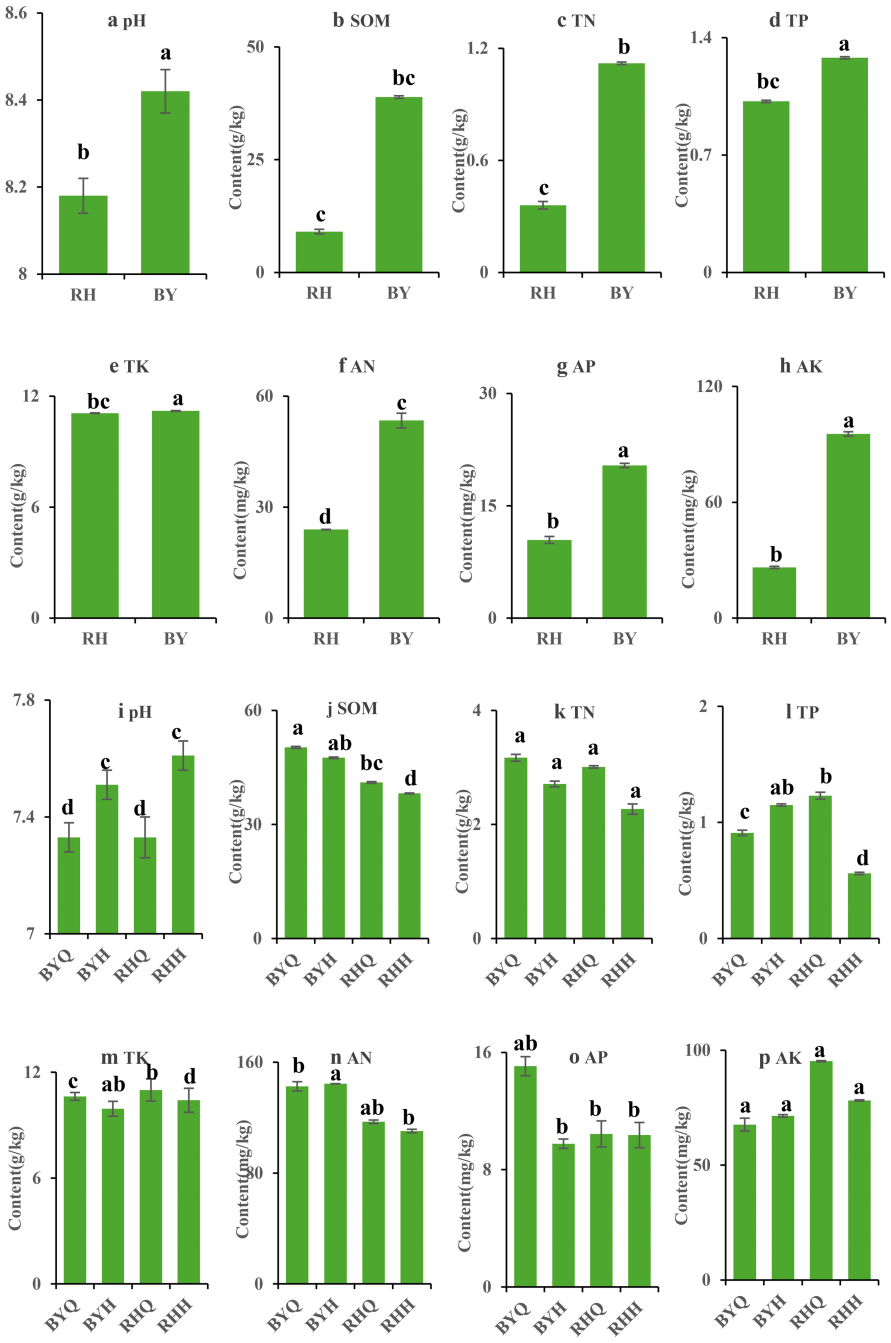
Basic physico-chemical properties of the inter-root soil of *Houttuynia cordata* Thunb. Note: ‘RH’ is Renhuai sample site; “BY” is Baiyun sample site; “RHQ” is derived from the soil before indoor cultivation of Fritillary in Renhuai; “RHH” is from the soil after indoor cultivation of fritillaria Renhuai; “BYQ” and “BYH” follow the same logic as above. The data shown are the mean and standard deviation of three replicates. *In situ* cultivation (A–H); indoor cultivation (I–P).

The physicochemical properties of soils under ISC and IC provide essential reference conditions for understanding the factors influencing rhizosphere microbial diversity in *H. cordata*. Significant differences were observed between the two cultivation methods (Fig. 3). While the soil properties before and after IC showed minimal variation, some changes were evident. Soils under ISC were slightly alkaline, whereas those under IC were closer to neutral. However, after cultivation, the soil pH increased, possibly due to root exudates from *H. cordata*. The contents of SOM, TN, and AN decreased, whereas TP, TK, AP, and readily AP remained relatively unchanged before and after IC, this may be attributed to the preferential uptake and utilization of carbon and nitrogen by *H. cordata*, with other nutrient elements being less affected. Overall, a comparative analysis of the two cultivation methods indicated that soil physicochemical properties were more significantly altered under IC > ISC.

### Rhizosphere bacterial community structure of *H. cordata* under different cultivation methods

**Figure 4:**
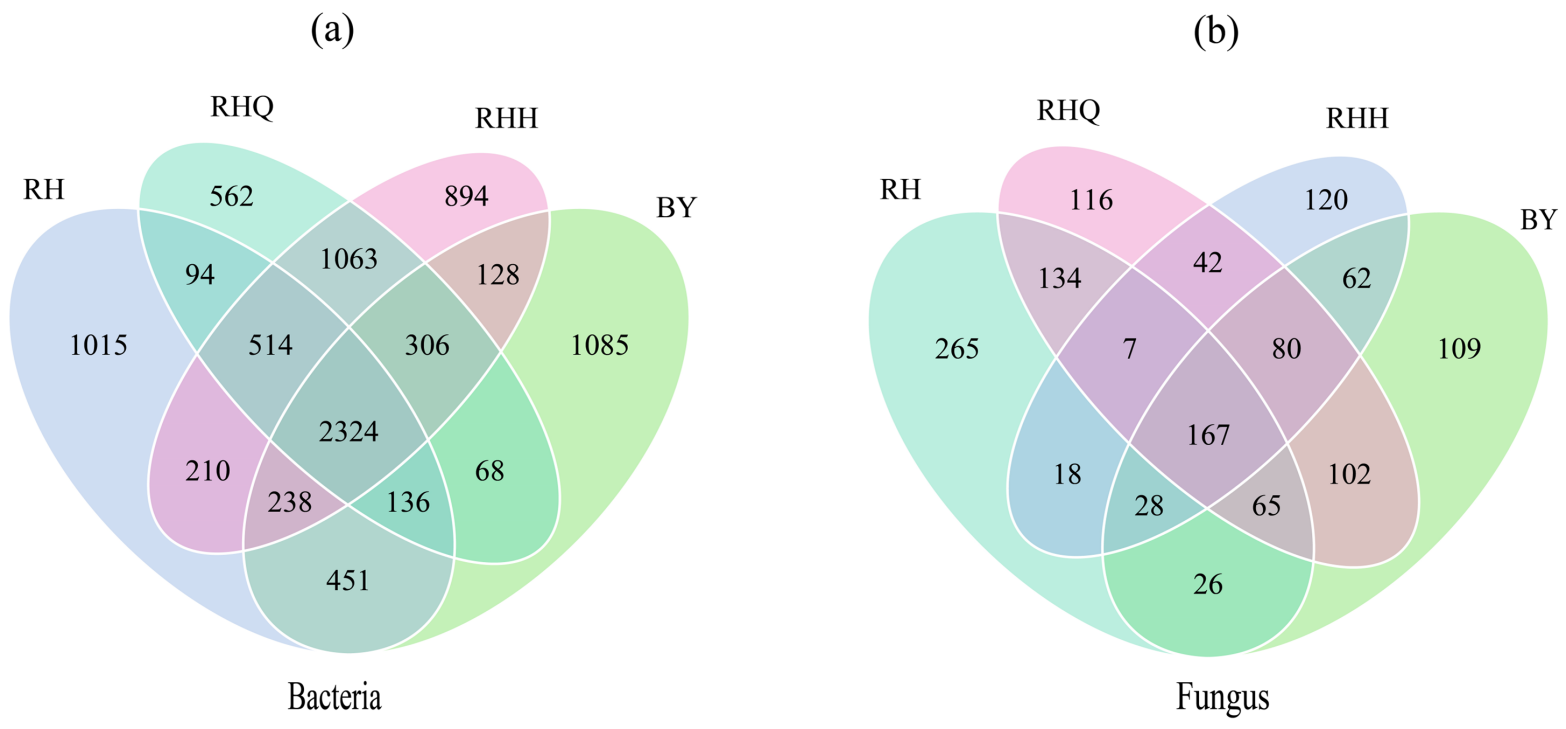
Venn diagram showing the number of bacteria (A) and fungi (B) at the level of OTUs in an inter-root soil sample of *Houttuynia cordata* Thunb. Each circle in the figure is coloured differently to indicate the number of species specific to the corresponding subgroup. The number of nuclei in the middle represents the number of OTUs common to all taxa. Note: “RH” denotes the rhizosphere soil of *H. cordata* collected from the sampling site in Renhuai city, Guizhou province; “BY” denotes the rhizosphere soil from the sampling site in Baiyun district, Guizhou province. “RHQ” represents the rhizosphere soil before indoor cultivation in Renhuai city, and “RHH” represents the rhizosphere soil after indoor cultivation in Renhuai city. The abbreviations “BYQ” and “BYH” follow the same naming convention for the respective samples from Baiyun district.

Under ISC, the Shannon index of rhizosphere soil bacteria showed little difference between the RH and BY sites. However, the Chao and Ace indices were greater at the RH site than at the BY site. Significant differences in bacterial community composition were observed between the two sites (*p* < 0.05), with OTU counts of 1015 at RH and 1085 at BY (Fig. 4). For IC, the Shannon, Chao, Ace, and Simpson indices increased after cultivation at the RH site, though the differences were not statistically significant (Table 2). This suggests that bacterial richness and diversity tend to be higher in the presence of plant roots. The number of OTUs at the RHQ and RHH sites under IC was 562 and 894, respectively (Fig. 4). Overall, the Shannon, Ace, and Simpson indices, as well as the total OTU count, were higher in ISC soils compared to IC. This suggests that ISC offers a more favorable environment for the rhizosphere bacterial community of *H. cordata*.

**Table 2:** Indicators of α-diversity of soil bacteria and fungi in the inter-root zone of *Houttuynia cordata* Thunb.

	Sample	Shannon	Chao	Ace	Simpson
Bacteria	RH	10.32 ± 0.08a	7,536.57 ± 224.23a	7,596.70 ± 145.30a	0.997 ± 0.00a
	BY	10.12 ± 0.13ab	6,661.70 ± 328.85b	6,717.93 ± 316.86b	0.997 ± 0.00a
	RHQ	9.95 ± 0.14b	7,506.30 ± 26.01a	7,529.37 ± 83.45a	0.996 ± 0.00b
	RHH	10.28 ± 0.05a	7,874.53 ± 150.99a	7,873.80 ± 183.45a	0.997 ± 0.00a
Fungi	RH	4.67 ± 0.14a	864.89 ± 59.47a	880.74 ± 49.00a	0.86 ± 0.02a
	BY	3.35 ± 0.52b	839.61 ± 53.05a	845.39 ± 17.54a	0.68 ± 0.13b
	RHQ	4.80 ± 0.51a	836.31 ± 43.71a	855.94 ± 25.24a	0.89 ± 0.04a
	RHH	5.20 ± 0.09a	722.64 ± 29.35b	716.48 ± 22.33b	0.92 ± 0.01a

**Notes.**

“RH” denotes the soil sampling site for *H. cordata* from the wild habitat in Renhuai city, Guizhou povince; “BY” denotes the plant sampling site for *H. cordata* from the wild habitat in Baiyun District. “BYQ” represents the soil before indoor cultivation in Baiyun district, and “BYH” represents the soil after indoor cultivation in Baiyun district; “RHQ” and “RHH” follow the same naming convention. Data in the table are presented as mean ± SE (standard error). Different lowercase letters within the same column indicate significant differences in soil physicochemical properties among sampling sites at *P* < 0.05.

**Figure 5:**
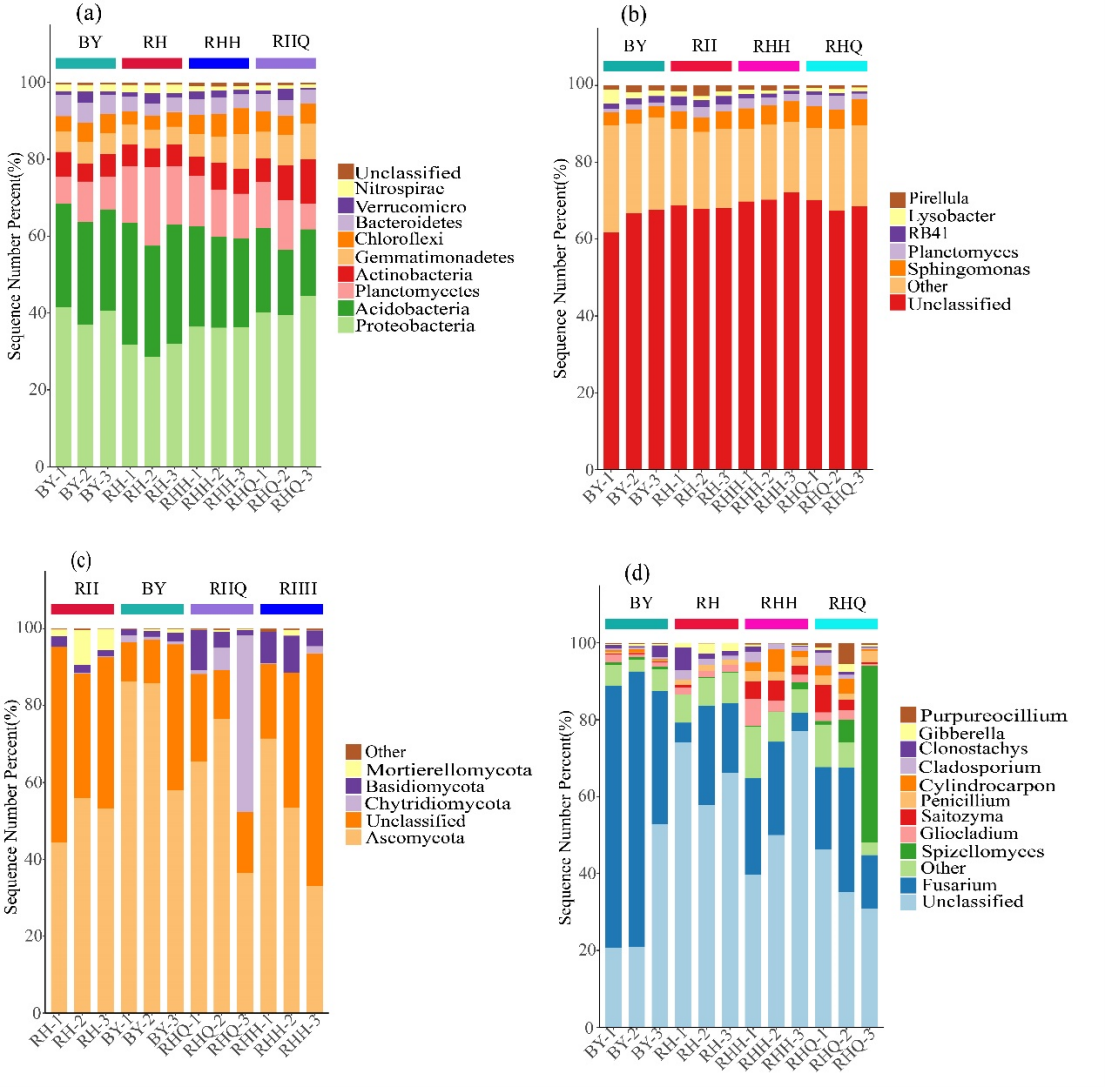
Taxonomic composition of the inter-root microbial community of *Houttuynia cordata* Thunb. ichneumonitis with stacked plots of relative abundance of bacteria at phylum (A) and genus (B) level, and of fungi at phylum (C) and genus (D) level. Note: “RH” denotes the rhizosphere soil of *H. cordata* collected from the sampling site in Renhuai city, Guizhou province; “BY” denotes the rhizosphere soil from the sampling site in Baiyun district, Guizhou province. “RHQ” represents the rhizosphere soil before indoor cultivation in Renhuai city, and “RHH” represents the rhizosphere soil after indoor cultivation in Renhuai city. The abbreviations “BYQ” and “BYH” follow the same naming convention for the respective samples from Baiyun district.

Soil microorganisms critically regulate disease suppression and promote plant growth. The dominant bacterial phyla in the rhizosphere of *H. cordata* under ISC and IC were Proteobacteria, Acidobacteria, Planctomycetes, and Actinobacteria, with relative abundances varying across samples (Fig. 5A). Proteobacteria dominated all samples, with relative abundances ranging from 39.65% in the RHQ IC sample to 29.99% in the RH ISC sample. Acidobacteria and Planctomycetes were more abundant in ISC, whereas Proteobacteria and Actinobacteria were more enriched in IC. Acidobacteria play key roles in nutrient cycling and organic matter decomposition in soil ecosystems. Renowned for broad soil pH tolerance, they drive key rhizosphere biogeochemical cycles—carbon, nitrogen, phosphorus, and sulfur. The high diversity of Acidobacteria across various soils and environments highlights their potential significance in plant-microbe interactions.

At the genus level, dominant bacterial genera numbered five in BY, 12 in RH, nine in RHQ, and eight in RHH under ISC and IC conditions (Fig. 5B). Compared to indoor cultivation, *Sphingomonas* and *Planctomyces* showed lower abundance in ISC, whereas *RB41* was more enriched.

These findings show that cultivation methods significantly shape the rhizosphere bacterial community of *H. cordata*, with ISC fostering more beneficial microbes than IC. Effects of different cultivation methods about the rhizosphere fungal communities associated with *H. cordata*.

Under ISC, Shannon, Simpson, Chao, and Ace indices for rhizosphere fungi were higher at RH than BY (Table 2). Under ISC conditions, the rhizosphere soil of *H. cordata* exhibited pronounced differences in fungal community composition, reflected by OTU counts of 265 at the RH site compared to 109 at the BY site (Fig. 4). Under IC, the Chao and Ace indices were higher than those under ISC, whereas the Shannon and Simpson indices showed the opposite trend (Table 2). Before and after sampling, the number of fungal OTUs in the RH site under IC was 116 and 120, respectively (Fig. 4). Collectively, these findings indicate that ISC markedly boosts fungal diversity in the rhizosphere soil of *H. cordata*.

Taxonomic resolution at the phylum level showed, Chytridiomycota, Ascomycota, Basidiomycota, Mortierellomycota, and unclassified fungi were the dominant groups in the rhizosphere of *H. cordata* under ISC. Ascomycota was the most abundant phylum, accounting for 51.16% (RH) and 76.73% (BY) of the total fungal community. Additionally, compared with the BY site, the RH site exhibited an increased relative abundance of Unclassified and Mortierellomycota, whereas the abundance of Ascomycota decreased (Fig. 5C).

These dominant fungal phylum were also identified under IC (Fig. 5C). The abundance of Chytridiomycota and Mortierellomycota increased following sampling, while Basidiomycota and unclassified taxa declined. Ascomycota stood out as the dominant phylum, representing 51.16% (RH), 59.49% (RHQ), and 52.63% (RHH) of the total fungal community in ISC and IC. Chytridiomycota was the second most abundant phylum, with the highest abundance observed in the RHQ site under IC, while it was almost absent in the RH site under ISC.

The top four genera at the genus level were *Fusarium*, *Spizellomyces*, *Gliocladium*, and *Saitozyma*. Among them, *Fusarium* accounted for 16.39% and 58.17% of the fungal communities in the RH and BY sites under ISC, respectively, whereas its relative abundance was 22.56% and 17.97% in the RHQ and RHH sites under IC (Fig. 5D). *Spizellomyces* was primarily detected in the RHQ site under IC, whereas *Gliocladium* and *Saitozyma* were mainly found in the RHH site under IC.

These findings indicate that different cultivation methods significantly alter the composition of the rhizosphere fungal community of *H. cordata*. ISC recruited more beneficial rhizosphere microbes compared to IC. However, the overall abundance of dominant fungi was lower in ISC. Notably, as commonly observed in bacterial community studies, Unclassified taxa accounted for 47.5% of the total fungal community, representing a substantial proportion that warrants further investigation.

### Heatmap analysis of rhizosphere soil microbial community distribution

**Figure 6:**
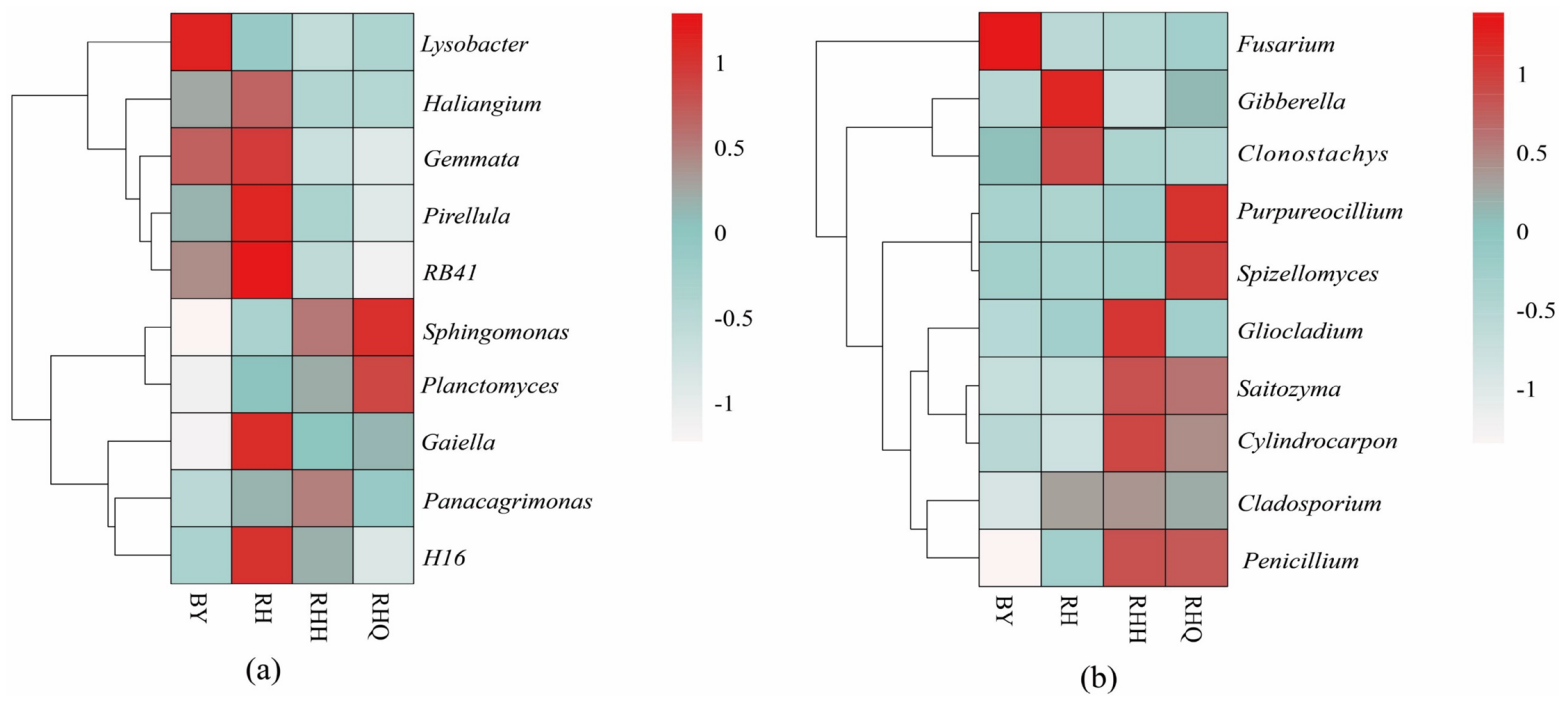
Heatmap of species abundance clustering of inter-root bacterial (A) and fungal (B) communities in *Houttuynia cordata* Thunb. Relationship between microbial community and soil physicochemical properties. Note: “RH” denotes the rhizosphere soil of *H. cordata* collected from the sampling site in Renhuai city, Guizhou province; “BY” denotes the rhizosphere soil from the sampling site in Baiyun district, Guizhou province. “RHQ” represents the rhizosphere soil before indoor cultivation in Renhuai city, and “RHH” represents the rhizosphere soil after indoor cultivation in Renhuai city. The abbreviations “BYQ” and “BYH” follow the same naming convention for the respective samples from Baiyun district.

Figure 6 presents clustering heatmaps of bacterial (Fig. 6A) and fungal (Fig. 6B) communities in the rhizosphere of *H. cordata*, revealing marked shifts in dominant microbial taxa across treatments. Under ISC, the dominant bacterial taxa in the RH site were *RB41*, *Gemmatimonas*, and *Pirellula*, while the predominant fungal genera were *Gibberella* and *Clonostachys*. In contrast, the BY site exhibited a distinct bacterial community, with *Lysobacter* as the dominant genus, whereas fungal diversity was relatively lower, with *Fusarium* being the main genus. The composition of dominant rhizosphere microbes appears to be shaped by geographic variation. Under IC, the dominant bacterial taxa in the RHQ site were *Planctomyces* and *Sphingomonas*, while the dominant fungal genera were *Purpureocillium* and *Spizellomyces*. In the RHH site, *Panacagrimonas* was the predominant bacterial genus, whereas the dominant fungal genera included *Gliocladium*, *Cylindrocarpon*, *Saitozyma*, and *Cladosporium*. These findings demonstrate that *H. cordata* cultivation significantly influences the structure of dominant soil microbial communities. Moreover, cultivation practices markedly shift the dominant bacterial and fungal taxa in the rhizosphere of *H. cordata*.

**Figure 7:**
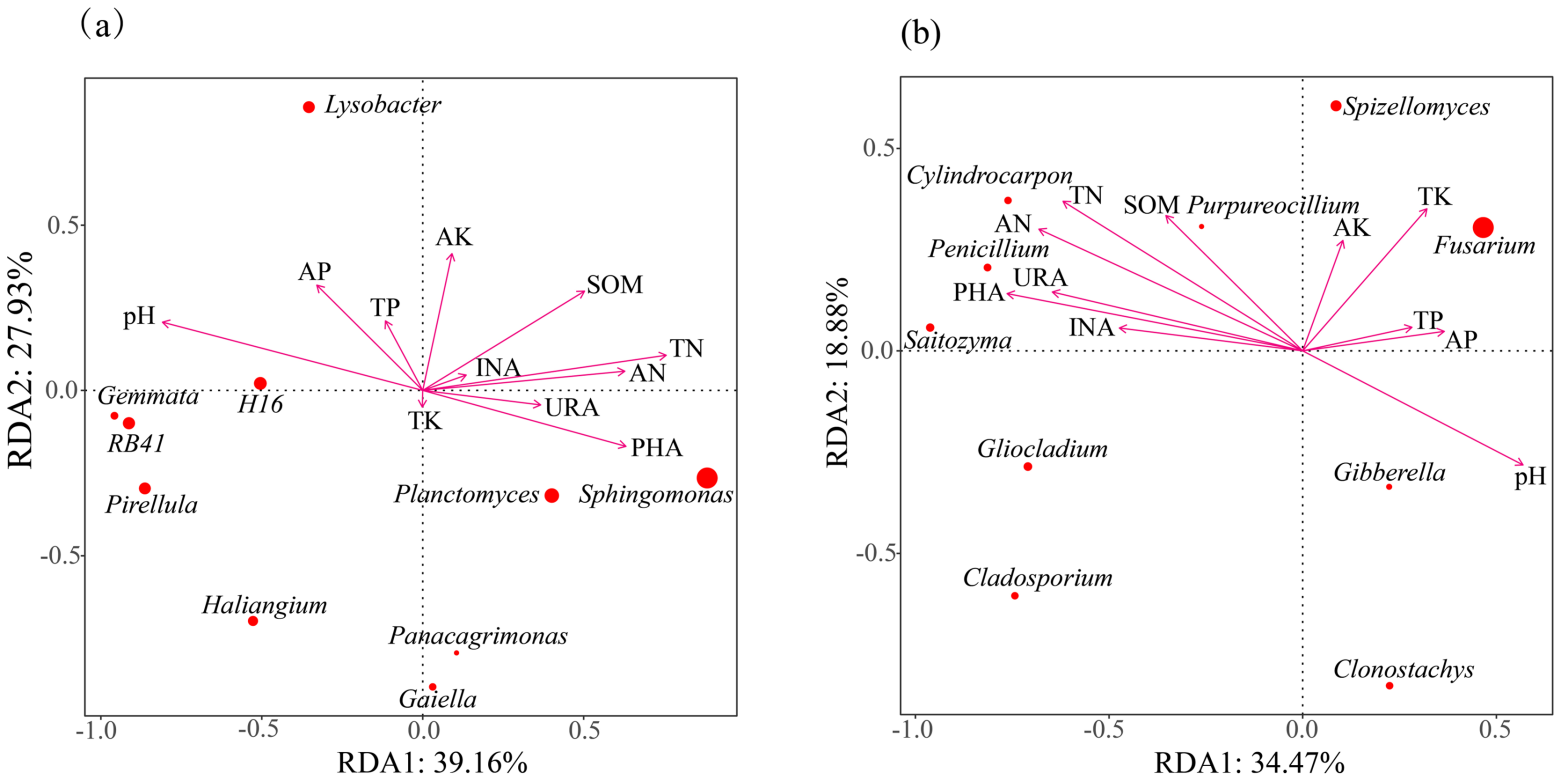
The correlation between the physicochemical properties of *Houttuynia cordata* Thunb. soil and the rhizosphere soil bacterial (A) and fungal (B) community structures of *H. cordata* was determined at the genus level. Environmental factors are represented by arrows; each point represents a genus, with larger points indicating higher scores.

RDA of the top 10 bacterial genera showed strong associations with soil environmental variables, with the first two axes explaining 39.16% (RDA1) and 27.93% (RDA2) of the total variation (Fig. 7A). Further correlation analysis identified soil pH, PHA, AN, TN, and SOM as key soil properties shaping the rhizosphere bacterial community of *H. cordata*. Among these factors, PHA exhibited the strongest influence on *Sphingomonas* and *Planctomyces*, whereas soil pH had a pronounced effect on *H16*, *Gemmata*, and *RB41*. A similar analysis was performed on the top 10 fungal genera (Fig. 7B), with RDA1 and RDA2 explaining 34.47% and 18.88% of the total variation, respectively. The major soil physicochemical properties influencing the rhizosphere fungal community included soil pH, PHA, AN, TN, SOM, TK, and urease (URA). Among these, TK had the greatest impact on *Fusarium*, while PHA and URA strongly influenced *Penicillium* and *Saitozyma*.

### Environmental driving factors of phenotypic variation in secondary metabolites of *H. cordata*

### Correlation analysis between secondary metabolites of *H. cordata* and soil physicochemical properties and soil enzymes

**Figure 8:**
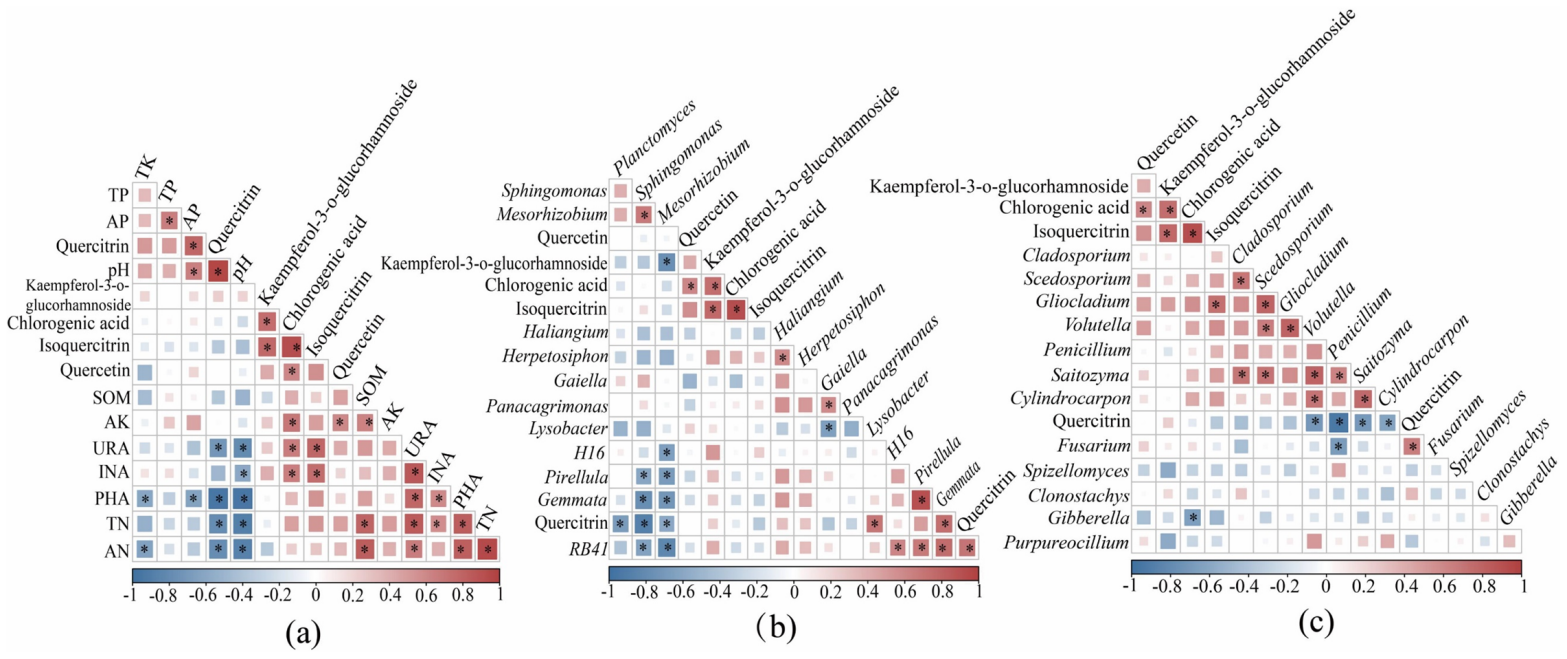
Heat map of correlation between secondary metabolic fractions of *Houttuynia cordata* Thunb. with soil physicochemical properties and soil enzymes (A), soil bacteria (B), fungi (C). Heat map of correlation between secondary metabolic fractions of *H. cordata* with soil physicochemical properties and soil enzymes (A); Heat map of correlation between secondary metabolic fractions of *H. cordata* with soil bacteria (B) and fungi (C); Significant positive correlations are shown as red circles, significant negative correlations are shown as blue circles, and ‘*’ indicates significant correlation (*p* < 0.05). Darker colours indicate higher correlation.

To identify potential environmental drivers, correlations were analyzed between *H. cordata* secondary metabolites and soil physicochemical properties and enzyme activities (Fig. 8A). quercitrin showed a significant positive correlation with AP and pH (*p* < 0.05), but was negatively correlated with URA, PHA, TN, and AN (*p* < 0.05). chlorogenic acid was positively correlated with AK, URA, and INA (*p* < 0.05). quercetin correlated positively with AK (*p* < 0.05), and isoquercitrin with URA and INA (*p* < 0.05).

### Correlation analysis between secondary metabolites of *H. cordata* and rhizosphere soil microorganisms

Plant secondary metabolites play a pivotal role in shaping plant—microbe interactions by acting as both signaling molecules and nutrient sources that influence microbial community composition and diversity. These metabolites influence soil feedback mechanisms and help structure rhizosphere microbial communities. We hypothesize that correlating rhizosphere microbial abundance with *H. cordata* secondary metabolites can reveal insights into microbial community composition and function.

To test this hypothesis, we conducted Spearman correlation analysis between the top ten microbial genera abundances and *H. cordata* secondary metabolites (Fig. 8B). quercitrin was significantly positively correlated with *Lysobacter*, *Gemmata*, and *RB41* (*p* < 0.05) but negatively correlated with *Planctomyces* (*p* < 0.05). kaempferol-3-O-glucorhamnoside showed a significant negative correlation with *Mesorhizobium* (*p* < 0.05). Among major fungal taxa (Fig. 8C), isoquercitrin correlated positively with *Gliocladium* (*p* < 0.05). quercitrin showed significant negative correlations with *Volutella*, *Penicillium*, *Saitozyma*, and *Cylindrocarpon* (*p* < 0.05), and a positive correlation with *Fusarium* (*p* < 0.05). Additionally, chlorogenic acid was negatively correlated with *Gibberella* (*p* < 0.05).

**Figure 9:**
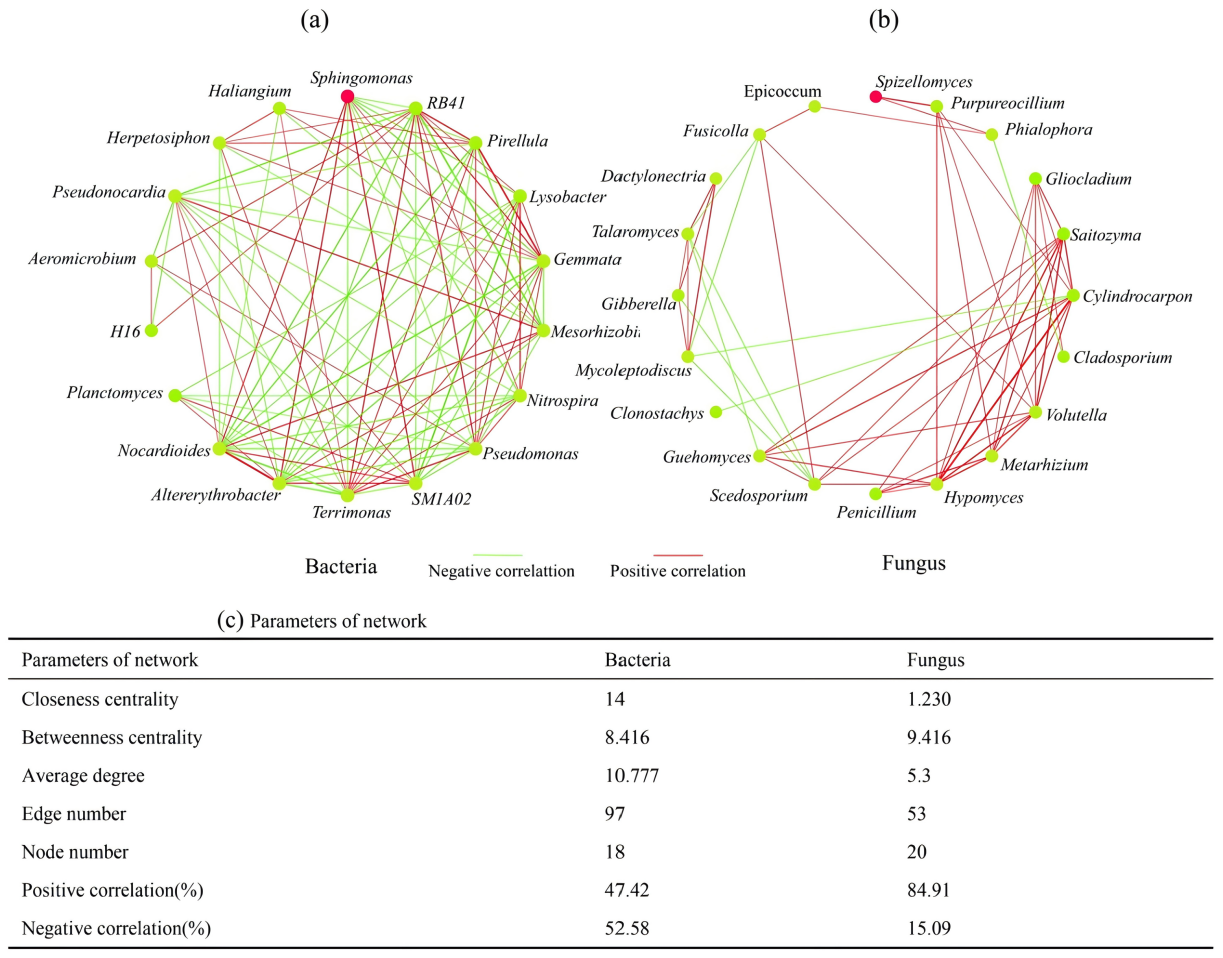
Network visualising the pattern of relationships between inter-root bacteria (A) and fungi (B) in *Houttuynia cordata* Thunb. at the genus level. (C) Network parameters. Each node represents a bacterial genus (A) and a fungal genus (B). Node size indicates the OTU richness of the gate. Edges are coloured according to the type of interaction. Positive correlations are marked with a red line and negative correlations with a green line.

The differences in interactions among rhizosphere microorganisms of *H. cordata* were confirmed through co-occurrence network analysis. In this study, network complexity was evaluated using closeness centrality, betweenness centrality, average degree, and the ratio of positive to negative correlations. Visually, the bacterial network in the rhizosphere of *H. cordata* (Figs. 9A–9C) exhibited a denser structure than the fungal network. The bacterial network had a higher closeness centrality (14) and average degree (10.77) compared to the fungal network (closeness centrality: 1.230; average degree: 5.3). However, the fungal network exhibited a higher betweenness centrality (9.416) than the bacterial network (8.416). Additionally, the bacterial network had 97 edges and 18 nodes, whereas the fungal network had 53 edges and 20 nodes. Within the rhizosphere microbial community of *H. cordata*, bacteria and fungi respectively exhibited 52.58% and 15.09% negative interactions.

## Discussion

In this study, the metabolite composition of *H. cordata* was significantly influenced by geographical factors. The content of phenolic compounds, including chlorogenic acid, rutin, kaempferol-3-o-glucorhamnoside, isoquercitrin, quercitrin, and quercetin, varied among different sampling sites. Previous studies have reported that quercetin content in *H. cordata* differs significantly with elevation (Wu et al., 2009). Similarly, quercitrin levels also exhibit significant regional variation (Yang et al., 2024). Moreover, wild *H. cordata* samples tend to have higher rutin and quercitrin concentrations than TC (Yang et al., 2024). These variations are likely attributed to environmental pressures associated with different geographical conditions, where *H. cordata* employs distinct defense mechanisms as adaptive survival strategies, particularly in high-altitude regions.

The genetic diversity within *H. cordata* populations is a key intrinsic driver of the variation in the types and content of its secondary metabolites. Existing studies indicate that populations of *H. cordata* from different regions of China exhibit significant genetic diversity. An ISSR-based analysis of 15 *H. cordata* populations in China revealed that genetic variation within populations was relatively low, while genetic differentiation among populations was high (Wei & Wu, 2012). Another study, utilizing SRAP markers to analyze *H. cordata* populations in the Huaihua region, revealed that the percentage of polymorphic bands (PPB) ranged from 53.66% to 85.35%, with an average heterozygosity of 0.07 to 0.23 and a Shannon’s index for the total population of 0.10 to 0.34, indicating a relatively high level of genetic variation (Jun et al., 2011; Jun et al., 2010). These genetic differences directly affect the activity and expression levels of key enzymes in the secondary metabolic pathways, leading to variations in the types and content of secondary metabolites synthesized by different *H. cordata* genotypes. While not conducted on *H. cordata* itself, research into other medicinal plants has demonstrated that distinct genotypes often display divergent metabolic profiles and concentrations, even when grown in the same controlled environment. The biosynthesis of key secondary metabolites in *H. cordata* such as volatile oils, flavonoids, and phenolic acids is governed by complex genetic networks, and genetic variations within these networks are ultimately responsible for the differences in the content of various medicinal components (Pradhan et al., 2023).

Studies have shown that chlorogenic acid and flavonoid contents in *H. cordata* peak at elevations of 1,400–1,500 m and 500–600 m respectively (Yang et al., 2010). In agreement with these findings, our results indicate that the concentrations of chlorogenic acid, kaempferol-3-o-glucorhamnoside, isoquercitrin, quercitrin, and quercetin were higher in the BY site (1,140–1,618.5 m) compared to the RH site (880 m). This consistency suggests that the metabolite composition of *H. cordata* is closely associated with environmental conditions, growth microhabitats, and the complexity of mountainous regions.

Overall, the variations in the content of different phenolic compounds in *H. cordata* differed among the three cultivation methods. Studies show that environmental stressors comprising low temperature, drought, and ultraviolet radiation induce the biosynthesis of secondary metabolites, especially phenolic compounds, in plants as a defense mechanism (Gallego-Narbón et al., 2024). The accumulation of phenolics varies among species depending on environmental cues. However, compared to TC under controlled conditions, IC was more favorable for the accumulation of phenolic compounds in *H. cordata*.

Among the three cultivation methods, the content of chlorogenic acid was highest in ISC, followed by IC and TC. Rutin was only detected in ISC. The content of quercetin was highest in IC. These differences indicate that the inherent environmental factors of each cultivation method have a profound impact on the metabolic characteristics of the plants. From a sustainable development perspective, it is crucial to optimize cultivation methods to enhance the accumulation of beneficial compounds without causing adverse environmental effects. If the content of specific phenolic compounds with high medicinal value can be maximized under IC conditions, it could reduce the large land use required by TC, thereby contributing to sustainability.

The phenotypic differences in secondary metabolites among the three cultivation methods were not driven by a single factor. In this study, the dominant microbial phyla in the rhizosphere of *H. cordata* included Proteobacteria, Acidobacteria, Planctomycetes, and Actinobacteria. This finding supports the widely accepted view that these phyla are common in soils globally (Li et al., 2023). Furthermore, in the fragile and challenging soil environment of ISC, *H. cordata* plants at the RHQ site exhibited a higher proportion of Actinobacteria. Studies indicate that Actinobacteria are more abundant in oligotrophic than in copiotrophic environments. This phylum is renowned for producing extracellular hydrolytic enzymes that break down recalcitrant organic matter like cellulose, chitin, and plant and animal residues. These processes not only enable microbial survival in nutrient-poor conditions but also enhance soil nitrogen cycling (Wu et al., 2021). Genomic studies suggest that many genes in this phylum are associated with nitrogen fixation and carbon cycling. However, our findings indicate a significant negative correlation between quercitrin and TN. Therefore, IC appears to promote higher quercitrin accumulation in *H. cordata*.

Across all samples, Ascomycota and Basidiomycota dominated fungal communities. These phyla, prevalent in soil fungi, are widely capable of degrading cellulose and lignin (Manici et al., 2024). Notably, Basidiomycota exhibited a significant positive correlation with soil C and N, with a substantial proportion of this phylum forming mycorrhizal associations with plant roots in soil (Zhu et al., 2022). Ascomycota tend to be more prevalent in harsh environments, whereas Basidiomycota thrive in nutrient-rich conditions. Furthermore, the relative abundance of Basidiomycota was higher under IC compared to ISC.

Several studies have reported a close association between fungal community diversity and potassium levels. Soil bacteria affect potassium solubility and availability, shaping the selection of taxa linked to potassium levels (Ding et al., 2024). In this study, AK content was higher in IC, with chlorogenic acid and quercetin showing significant positive correlations with AK (*p* < 0.05). Notably, kaempferol-3-O-glucorhamnoside and Mesorhizobium exhibited significant negative correlations with AK (*p* < 0.05) but were positively correlated with chlorogenic acid content (*p* < 0.05).

Soil pH is widely regarded as a key determinant of soil properties, as it influences nutrient solubility and microbial metabolism (Naz et al., 2022). High rainfall and organic matter decomposition increase soil acidity (Ni et al., 2021; Ni et al., 2022). Soil pH is a key driver of bacterial community structure in agricultural soils, influencing it both directly and indirectly (Che et al., 2022). It chiefly governs bacterial distribution and balances deterministic and stochastic processes in community assembly (Ni et al., 2021). Soil pH directly shapes the relative abundance of dominant bacterial taxa by affecting microbial growth tolerance under varying redox conditions. As a key driver of microbial community structure, it differentially influences bacterial and fungal communities. Some dominant taxa, including Acidobacteria and Proteobacteria, were negatively correlated with pH (Tan et al., 2023), whereas Actinobacteria and Bacteroidota exhibited a positive correlation with pH (Ni et al., 2021). These findings indicate bacterial communities are more sensitive to soil pH fluctuations than fungi, consistent with previous studies showing that bacteria is narrower growth tolerance makes them more responsive to pH changes (Ni et al., 2021).Our findings suggest that fungi contribute to the degradation of organic matter into low-molecular-weight metabolites in *H. cordata* cultivation, thereby altering the rhizosphere microbial community structure. In this context, AK has been identified as a major factor shaping microbial community (Tan et al., 2023). This aligns with our results, which indicate that AK is a key driver of fungal community structure. Additionally, previous studies have reported that *H. cordata* growth is favored under mildly acidic and moist conditions, which is consistent with our findings.

Soil physicochemical properties were also significantly altered by cultivation methods, particularly between ISC and IC. Soil under ISC were slightly alkaline, while those under IC were closer to neutral initially, but pH increased after cultivation, possibly due to root exudates. Critically, the contents of SOM, TN, and AN decreased under IC, potentially due to preferential uptake by *H. cordata*, while TP, TK, AP, and readily available phosphorus remained relatively unchanged. The more significant alterations in soil physicochemical properties under IC compared to ISC highlight the need for sustainable soil management practices. If IC leads to nutrient depletion or adverse pH changes, long-term application would require strategies like nutrient supplementation or soil amendments to maintain soil fertility and productivity, thus ensuring agricultural sustainability.

Isoquercitrin exhibited a significant positive correlation with URA and INA (*p* < 0.05), as well as with *Gliocladium* (*p* < 0.05). However, URA had the greatest impact on *Saitozyma*, which was predominantly present under laboratory incubation compared to ISC. Therefore, IC facilitates the accumulation of isoquercitrin in *H. cordata*.

## Conclusions

Compared to ISC, IC significantly improved rhizosphere soil fertility and health in *H. cordata*. Additionally, IC enhanced the abundance of beneficial soil microorganisms, the genera Saitozyma, Lysobacter, Gemmata, and Penicillium were significantly associated with plant growth. Numerous biocontrol and growth-promoting microbes likely support the healthy growth of *H. cordata* and enhancing its adaptability to different environments.

Soil pH, URA, AK, and TN were identified as key factors influencing the accumulation of phenolic compounds in *H. cordata*, along with the makeup and organization of the soil microbial community. Different cultivation methods had distinct effects on the rhizosphere microbial community structure. Taxonomic analysis identified Proteobacteria, Actinobacteria, and Acidobacteria as dominant bacterial phyla, and Ascomycota and Basidiomycota as dominant fungal phyla. At the taxonomic level of genus, *Sphingomonas* was the most abundant bacterial genus, while *Fusarium*, *Spizellomyces*, *Gliocladium*, and *Saitozyma* dominated the fungal community. Some of these taxa function as biocontrol agents, supporting the growth and health of *H. cordata*.

Structural analysis indicated that pH, URA, TK, and TN were key determinants of microbial community composition and also major drivers of the accumulation of *H. cordata* phenolic compounds, including quercitrin, kaempferol-3-O-glucorhamnoside, isoquercitrin, and chlorogenic acid. Notably, compared to ISC, IC was more conducive to the accumulation of phenolic compounds in *H. cordata*.

In summary, our study sheds light on the rhizosphere microbial communities of *H. cordata* under different cultivation methods and their role in shaping community structure. Additionally, we identified pH, URA, TK, and TN as key environmental drivers influencing the phenotypic variation in the secondary metabolites of *H. cordata*. Our findings suggest that laboratory incubation creates a favorable rhizosphere soil environment for *H. cordata* and positively contributes to the accumulation of phenolic compounds. This research offers a new perspective on the complex interactions between rhizosphere microorganisms and plants under different cultivation conditions and provides a foundation for enhancing the sustainability of agricultural production.

##  Supplemental Information

10.7717/peerj.20797/supp-1 Supplemental Information 1 Original data on the basic properties of soil, enzymes, and phenolic substances

## Additional Information and Declarations

### Data Availability

The following information was supplied regarding data availability: The raw measurements are available in the Supplemental Files. The data is available at Figshare: Song, fangmei (2025). Effects of Different Cultivation Models on the Rhizosphere Microbial Community of *Houttuynia cordata* Thunb.sequence.zip. figshare. Dataset. https://doi.org/10.6084/m9.figshare.29967286.v1
Soil microbial sequencing data are available under the NCBI BioProject: PRJNA1304453.
